# Behavioral dysregulation at work: A moderated mediation analysis of sleep impairment, work-related stress, and substance use

**DOI:** 10.3934/publichealth.2025018

**Published:** 2025-03-12

**Authors:** Francesco Marcatto, Donatella Ferrante, Mateusz Paliga, Edanur Kanbur, Nicola Magnavita

**Affiliations:** 1 Department of Life Sciences, University of Trieste, Trieste, Italy; 2 Institute of Psychology, Faculty of Social Sciences, University of Silesia, Katowice, Poland; 3 Department of Psychology, Ibn Haldun University, Istanbul, Türkiye; 4 Post-graduate School of Occupational Health, Università Cattolica del Sacro Cuore, Rome, Italy

**Keywords:** work-related stress, sleep impairment, counterproductive work behaviors, substance use, moderated mediation analysis

## Abstract

**Background:**

Sleep impairment and work-related stress are common issues that influence employee well-being and organizational outcomes. Impaired sleep depletes cognitive and emotional resources, increasing stress and the likelihood of counterproductive work behaviors directed toward the organization (CWB-O). This cross-sectional study, guided by the conservation of resources (COR) theory, explores the relationships between impaired sleep, work-related stress, and CWB-O, considering substance use as a dysfunctional coping strategy.

**Methods:**

A sample of 302 Italian employees completed an online survey. Sleep impairment was assessed using the Insomnia Severity Index, work-related stress was assessed with the Perceived Stress Scale, CWB-O was assessed with the Counterproductive Work Behavior Checklist, and substance use as a coping strategy was assessed using the Brief COPE. A moderated mediation model was tested to examine the indirect effects of sleep impairment on CWB-O via work-related stress, with substance use moderating both the sleep–stress and stress–CWB-O relationships.

**Results:**

The results supported the hypothesis that the relationship between sleep impairment and CWB-O is mediated by work-related stress. Sleep difficulties significantly increased work-related stress, which in turn led to higher levels of CWB-O. Substance use did not moderate the relationship between sleep and work-related stress. It did, however, significantly moderate the relationship between work-related stress and CWB-O, with higher levels of substance use amplifying the impact of stress on behavioral dysregulation.

**Conclusion:**

This study contributes to our understanding of how impaired sleep, work-related stress, and substance use interact to influence deviant behaviors at work. The findings align with COR theory, highlighting the role of resource depletion and dysfunctional coping in workplace behavior, and suggest that organizational interventions should also consider programs aimed at improving sleep quality and addressing substance use to reduce the likelihood of deviant behaviors at work.

## 1. Introduction

The organization of work—the complex series of factors that govern the way in which work is divided, controlled, and coordinated—requires close interdependence among workers [1]. Work organization is one of the factors that allow a company to compete in the market. According to the literature, good organization is associated with higher worker satisfaction and well-being, which in turn positively impacts productivity, innovation, and overall organizational success, which are key factors in maintaining a competitive edge [2],[3]. Conversely, organizational problems are linked to physical and psychological health issues among workers, potentially leading to reduced performance and absenteeism [4]–[6]. Evidence from the literature indicates that workers' behavior significantly influences the organization of work. For example, collaborative behaviors among employees can enhance coordination and efficiency, while counterproductive behaviors may disrupt workflow and hinder organizational goals [7],[8].

Counterproductive work behaviors directed toward the organization (CWB-O) represent a serious form of behavioral dysregulation consisting of intentional actions that directly conflict with organizational goals, such as sabotage, theft, or deliberate inefficiency [9]. Research suggests that these actions can be interpreted as maladaptive reactions and impaired functioning resulting from factors such as sleep impairment, work-related stress, and dysfunctional coping mechanisms, including the use of substances like alcohol or drugs to manage stress or fatigue [10],[11].

Sleep problems, which include sleep disorders and sleep deprivation, can dramatically disrupt an individual's daily functioning, general health, and work performance [12],[13]. Sleep disorders are a heterogeneous group of conditions that are often underdiagnosed and undertreated, with the most common being obstructive sleep apnea and insomnia [14],[15]. Sleep deprivation is usually intentional or due to circumstances that prevent someone from getting enough sleep, such as long work hours, shift work, staying up late, caregiving, or excessive use of electronic devices [16],[17]. Research shows an elevated incidence of sleep deprivation in Western countries, with one out of three persons reporting sleep problems [18].

Sleep impairment, particularly difficulties falling asleep and/or staying asleep, is common among workers across multiple disciplines. The health, manufacturing, and service sectors are among the most at-risk industries [19]. Sleep impairment negatively impacts workers' health, work performance, and work safety [15],[20]–[23], making it a primary concern for both employees and employers [24]. Lack of sleep and poor sleep quality are known to heighten emotional reactivity, decrease attention, and impair decision-making [25]–[29], ultimately lowering employees' ability to work effectively.

One of the most important factors associated with sleep problems is work-related stress since it interferes with both the quality and quantity of sleep [21],[30],[31]. Work-related stress is defined as a set of behavioral, emotional, cognitive, and physiological responses to unpleasant aspects of work, characterized by elevated levels of arousal and distress [32]. Similarly to sleep impairment, work-related stress is a well-known risk factor for different negative outcomes, such as mental and physical illnesses, burnout, reduced job satisfaction, and increased turnover intention [33]–[37].

Risk factors for work-related stress include excessive job demands [38], shift work [39], extended working hours [40], workplace violence [41],[42], and lack of adequate support and bad relationships at work [43]. According to scientific literature, impaired sleep is both a consequence and a risk factor for work-related stress. Indeed, employees who experience higher job stress suffer more frequently from insomnia [44], and working in an unfavorable psychological environment is known to double the risk of suffering from insomnia [45]. On the other hand, sleep-deprived employees may find it difficult to cope with job demands and are therefore more vulnerable to work-related stress [14],[46],[47]. This circular relationship suggests that impaired sleep and work-related stress are interconnected, with each amplifying the effect of the other [22],[31],[48] in a vicious cycle that affects both employee health and organizational effectiveness [49]–[51].

CWB-O can be seen as an organizational-level consequence of this interplay between sleep and work-related stress [10]. Indeed, employees experiencing high levels of stress and sleep deprivation might be more likely to engage in such behaviors as a form of retaliation, further worsening their negative impacts on the organization [52]–[54].

In addition to the effects of impaired sleep and work-related stress on CWB-O, this study also considers the role of substance use as a dysfunctional coping mechanism. Substance use may worsen the negative effects of inadequate sleep and occupational stress, increasing the likelihood of behavioral dysregulation [55],[56]. This study aims to investigate the intricate relationships among these constructs, with a focus on substance use as a dysfunctional coping strategy that moderates these relationships, a dimension scarcely explored in prior research. By shedding light on how these interactions affect employee voluntary behavior aimed at harming the organization and its outcomes, this study also aims to provide insights for developing strategies to mitigate these risks and promote healthier and more productive workplaces.

### 1.1. Theoretical framework

#### 1.1.1. The conservation of resources theory

The conservation of resources (COR) theory provides a comprehensive framework for understanding how individuals react to stress, especially regarding resource gain and loss [57]. According to COR theory, people are motivated to acquire, protect, and maintain resources necessary for their well-being and protection against possible resource loss. These resources include material possessions (e.g., money), personal characteristics (e.g., self-esteem), and energy (e.g., attention). People experience stress when there is a threat of resource loss, an actual loss of resources, or insufficient resource gain after an investment of resources [58].

In the context of this study, COR theory is especially relevant for explaining the relationships among impaired sleep, work-related stress, and CWB-O. Since sleep is essential for preserving overall health, emotional stability, and cognitive function, it can be viewed as a critical personal resource [59]. This vital resource is exhausted by poor or interrupted sleep, which increases a person's susceptibility to stress [54]. Therefore, according to this theory, when employees do not get enough sleep, their ability to handle their job demands may be diminished, which could result in an increase in work-related stress [60].

This depletion of resources, as described by COR theory, also has implications for employees' behavior at work. People who experience a decrease in sleep-related resources may become more stressed and engage in maladaptive behaviors, like CWB-O, to deal with or react to this perceived loss of resources [56],[61].

Moreover, COR theory contributes to the understanding of how dysfunctional coping strategies, such as substance use, moderate the relationship between impaired sleep, work-related stress, and CWB-O [62]. Substance use can be viewed as a maladaptive and avoidant [63] attempt to cope with stress and lack of sleep and to replenish lost resources [64],[65]. However, instead of restoring resources, substance use further depletes them by worsening sleep quality and increasing stress, thus leading to a resource-loss spiral [58]. Therefore, engaging in substance use as a coping strategy could exacerbate the strength of the vicious cycle in which lack of sleep leads to further work-related stress, ultimately increasing the likelihood of behavioral dysregulation in the form of counterproductive behaviors at work [9],[56],[66].

In summary, COR theory offers a useful lens to understand the connections among impaired sleep, work-related stress, and counterproductive work behaviors. By framing these constructs in terms of resource loss and conservation, COR theory highlights the importance of managing sleep and stress in order to prevent the escalation of negative behaviors in the workplace.

#### 1.1.2. Impaired sleep and work-related stress

Sleep is an essential resource that restores the emotional and cognitive reserves needed for effective functioning [59],[66]. Lack of sleep prevents fully replenishing these reserves, leading to a reduced ability to handle job demands [67],[68], decreased job performance, and increased sick leaves and accidents [19]. Therefore, employees suffering from sleep impairment are more likely to perceive job demands as overwhelming and less likely to feel confident in their ability to meet them. As employees feel less capable of coping with their duties, this negative appraisal of job demands can increase stress levels [24],[69]. This, in turn, can lead to further sleep disruption, perpetuating a cycle of resource loss that reduces both individual well-being and workplace performance, lowering job satisfaction and increasing the likelihood of burnout and engaging in counterproductive work behaviors [70],[71].

Although there is a reciprocal relationship between work-related stress and sleep problems, with each influencing the other [22],[31],[48], longitudinal research indicates that, over time, sleep impairment is more likely to predict increases in work-related stress than work-related stress is to predict sleep problems [21]. This finding emphasizes the critical role of sleep as a precursor to stress, rather than just a consequence of it, also highlighting the necessity of treating sleep issues to prevent work-related stress from getting worse.

#### 1.1.3. Work-related stress and counterproductive work behaviors

Work-related stress is a significant predictor of counterproductive work behaviors directed toward the organization (CWB-O), which consist of intentional actions of employees that infringe on an organization's interests, violate its rules, and hurt the organization itself [10],[72]. It includes actions such as taking longer breaks, reducing effort, or even deliberately harming organizational assets. High levels of stress can impair employees' ability to manage emotions, maintain self-control, and follow organizational norms, leading to an increased likelihood of engaging in these dysfunctional behaviors [52],[73].

Depletion of psychological and emotional resources represents one of the mechanisms through which work-related stress contributes to CWB-O [10],[60]. Stress depletes the mental and emotional energy needed to respond effectively to job demands and challenges. Consequently, as stress levels rise, employees can experience feelings of overload, frustration, or even resentment, and respond by engaging in behaviors that harm the organization. Indeed, these actions can be interpreted as an attempt to recover a sense of control or balance in response to the stress caused by resource depletion. For example, an employee who feels overwhelmed by excessive job demands and exhausted due to lack of sleep may decide to sabotage organizational processes or violate workplace norms. These actions could include taking longer breaks than allowed or taking sick leaves under false pretenses, either as a form of retaliation or as a misguided effort to conserve the remaining personal resources [74].

Moreover, work-related stress can also evoke negative emotions and attitudes and thus distort employees' perception of their workplace, increasing the likelihood that they will view it as unjust or even hostile [75],[76], fostering feelings of dissatisfaction and disengagement, which could further increase the likelihood of CWB-O [56],[77]. Indeed, employees who feel unfairly treated may justify deviant actions as retaliation against perceived injustice [78].

High levels of stress can also impair decision-making and self-regulation, leading to impulsive or short-sighted behaviors that employees might not otherwise consider [79],[80]. When employees are not experiencing high levels of stress or are working in a supportive environment, they are usually capable of following workplace norms and regulations, but when overwhelmed by stress, they are less capable of controlling their impulses [81]. As a consequence, they are more prone to acts of retaliation or sabotage, which hurt in the long run both the organization and the employee, even though they may seem satisfactory in the short term [79].

In summary, by depleting personal resources, increasing negative perceptions of the workplace, reducing self-regulation, and impairing decision-making, work-related stress contributes to the occurrence of CWB-O.

#### 1.1.4. The moderating role of substance use

Coping mechanisms are critical in determining which behaviors will be adopted to address aversive situations [82]. The use of substances such as alcohol, drugs, or other psychoactive agents can serve as a coping strategy that people could use to manage stress and discomfort [64]. Differently from functional coping strategies, such as seeking social support [83], substance use may provide short-term relief only, since it does not address the underlying causes, and often leads to further psychological and physiological issues, thus exacerbating the very problems it was meant to alleviate [84]. Therefore, we believe that substance use could play a relevant role in the relationships between impaired sleep, work-related stress, and CWB-O, leading to a higher likelihood of engaging in deviant behaviors at work.

First, substance use may strengthen the relationship between sleep difficulties and work-related stress. Lack of sleep already depletes cognitive and emotional resources, leaving individuals vulnerable to stress. When substances such as alcohol, cannabis, benzodiazepines (BZD), or opioids are used as a coping mechanism, they further disrupt sleep quality, thus increasing resource depletion [85]–[87]. Research has shown that alcohol and cannabis use can increase sleep latency and disrupt the normal architecture of REM and NREM sleep [88], while chronic BZD use reduces slow-wave brain activity [89], and long-term opioid use leads to increased awakenings, decreased total sleep time, and diminished sleep efficiency [87]. These effects aggravate the vicious cycle where both sleep quality and stress levels continue to deteriorate.

Second, substance use may also moderate the relationship between work-related stress and CWB-O. Substance use, including alcohol and stimulants, intensifies the negative emotions associated with stress, such as frustration, anxiety, and irritability [90]. It also impairs decision-making [91] and self-regulation [92], therefore increasing the likelihood that stressed employees will act out against their organization. For example, alcohol consumption has been shown to heighten emotional reactivity and reduce impulse control, while stimulants such as cocaine impair long-term decision-making and increase risk-taking behaviors [90]–[92]. This combination of sleep disruption, heightened stress, and impaired self-regulation could represent a strong risk factor for the onset of deviant behaviors such as CWB-O.

### 1.2. Hypotheses development

Drawing upon the theoretical framework and the relationships between the constructs described in the previous sections, we propose a set of hypotheses about the connections among sleep, work-related stress, and CWB-O, also considering the moderating role of substance use.

As previously noted, according to COR theory, sleep problems deplete a critical personal resource, restorative sleep, essential for preserving cognitive, emotional, and behavioral functioning. Moreover, the lack of sleep impairs judgment, reduces emotional resilience, and heightens irritability, which can increase the likelihood of engaging in deviant behaviors at work. This relationship between impaired sleep and CWB-O, however, may be mediated by work-related stress. Due to the cognitive and emotional resource depletion caused by impaired sleep, employees are more likely to struggle in managing job demands and interactions at work, therefore being more susceptible to stress. This increased stress, in turn, can lead to higher levels of CWB-O as employees struggle to manage their duties effectively. Therefore, we hypothesize:

*H1: Impaired sleep is a significant predictor of CWB-O, but this relationship is mediated by work-related stress*.

Substance use, as a dysfunctional coping strategy, can amplify the negative effects of both impaired sleep and work-related stress. When employees take substances to relieve the discomfort caused by sleep problems and stress, they deplete their cognitive and emotional resources even more. This additional resource depletion heightens the negative impact of lack of sleep on stress. Moreover, the impairment in decision-making and self-regulation caused by substance use makes it more difficult for individuals to manage their stress and, therefore, increases the likelihood of engaging in CWB-O. Thus, we hypothesize:

*H2a: The relationship between impaired sleep and work-related stress is moderated by the dysfunctional coping strategy of substance use*.

*H2b: The relationship between work-related stress and CWB-O is moderated by the dysfunctional coping strategy of substance use*.

**Figure 1.**
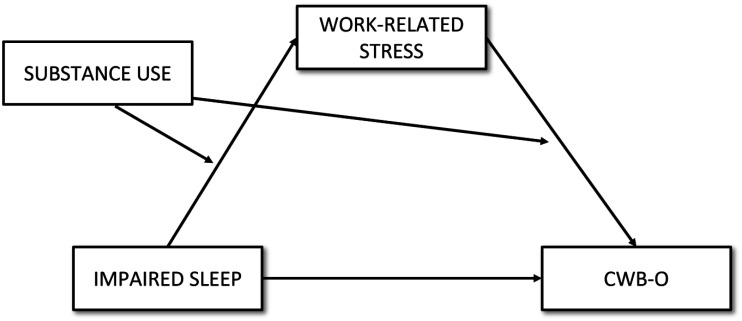
A conceptual model for testing moderated mediation.

These hypotheses are conceptualized in the model presented in Figure 1.

## 2. Materials and methods

### 2.1. Participants and procedure

A total of 302 participants were recruited through Prolific©, an online survey platform. The source population comprised Italian adult employees registered on the Prolific© panel. The choice of the Prolific© platform for case recruitment was made on the basis of a careful assessment of data security conditions. The platform in fact acts as a data controller, leaving the researcher with the responsibility for complying with data protection laws. Using the platform's filtering system, we identified and invited eligible participants who met the following inclusion criteria: being at least 18 years old, currently employed, residing in Italy, and having Italian as their primary language. There were no additional exclusion criteria. To ensure that participants were actually employed, they had to confirm their current employment status on the first page of the questionnaire. Participants received the link to the online questionnaire developed specifically for this study via Prolific© and were financially compensated with £0.75 for their participation. The study used a cross-sectional design and was approved by the Ethics Committee of the University of Trieste, Italy (Minutes No. 5, dated May 27, 2024), and was conducted in accordance with the Helsinki Declaration. Written informed consent, which emphasized the confidentiality of the data and the participant's right to withdraw from the study at any time, was provided on the first page of the questionnaire, where participants were asked to click “I consent” to proceed with the study. Data was collected between May 28, 2024, and May 30, 2024.

### 2.2. Measures

Participants were asked to complete a questionnaire that included the validated psychometric tools described below.

*Insomnia Severity Index*. The Insomnia Severity Index (ISI) [93] is a self-report instrument designed to evaluate perceived sleep difficulties. It consists of seven items evaluating the subjective symptoms and daytime consequences of insomnia, with responses on a 5-point Likert scale (from 0 to 4). The total score, which ranges from 0 to 28, is derived by summing the individual item responses, with higher scores indicating greater perceived sleep difficulties. In this study, we employed the Italian version of the tool [94]. Cronbach's alpha in the current study was 0.87.

*Perceived Occupational Stress Scale*. The Perceived Occupational Stress Scale (POS) [95] is a four-item psychometric tool that measures the level of stress that individuals experience at work. Each item is rated on a 5-point Likert scale (from 1 to 5). The total score, which ranges from 1 to 5, is calculated by averaging the individual item responses, with higher scores reflecting a higher level of perceived stress at work. Cronbach's alpha in the current study was 0.87.

*Counterproductive Work Behavior Checklist*. The Italian version of the Counterproductive Work Behavior Checklist [96] is a 45-item questionnaire that measures the frequency of two types of CWB: those directed toward the organization (CWB-O, e.g., “Purposely did your work incorrectly”) and those directed at individuals (CWB-I, e.g., “Refused to help someone at work”). Items are answered on a 5-point scale (from 1 to 5). For each subscale, a total score, which ranges from 1 to 5, is calculated by averaging the individual item responses, with higher scores indicating a higher frequency of CWB. In this study, only the CWB-O subscale was used, with a Cronbach's alpha of 0.89.

*Brief COPE Scale*. The Italian Brief COPE [97] is a 21-item scale used to assess six coping strategies that individuals might use in stressful situations, namely activation, deactivation, social support, humorous reframing, religious/spiritual reliance, and substance use. Items are rated on a 4-point Likert scale (from 1 to 4). For each subscale, a total score, which ranges from 1 to 4, is calculated by averaging the individual item responses, with higher scores indicating greater use of that coping strategy. In this study, only the substance use subscale (COPE-SUBST) was used, with a Cronbach's alpha of 0.92.

### 2.3. Data analysis

First, standard descriptive statistics and correlational analyses (using the Pearson correlation coefficient) were conducted to explore the relationships among the variables. Then, the hypothesized moderated mediation model (Figure 1) was tested in a single model, using the Jamm module v. 1.2.1 for Jamovi. This approach allowed for the assessment of the significance of the indirect effects of impaired sleep on CWB-O through work-related stress across different levels of the moderator (substance use). To evaluate the mediational relationship (ISI–POS–CWB-O) at varying levels of substance use, analyses were conducted at three specific levels of the moderator: low (1 SD below the mean COPE-SUBST score), average (mean COPE-SUBST score), and high (1 SD above the mean COPE-SUBST score). Mediation (H1) was considered to occur when a significant indirect effect was observed at the average level of COPE-SUBST. The difference in indirect effects across the low, average, and high levels of COPE-SUBST was used to assess the presence of moderated mediation (H2a and H2b), indicating whether the strength of the mediation effect varied depending on the level of substance use. A p-value < 0.05 was used as the threshold for statistical significance. No formal a priori sample size calculation was conducted for this study. However, with a sample size of 302 participants, the study had more than adequate statistical power to detect medium-sized effects, as per standard guidelines for regression-based analyses in behavioral research [98]. Moreover, a post hoc power analysis for the indirect path in the moderated mediation model was conducted using the online MedPower tool [99]. With a sample size of 302 participants, the power to detect the indirect effect was estimated to be 0.84, indicating adequate statistical power for the analysis.

Missing data were minimal (approximately 1%), and listwise deletion of cases was used.

## 3. Results

Demographic and work-related data of the study sample are reported in Table 1.

**Table 1.** Demographic and work-related characteristics of the study sample.

Variable	n (%)/mean (SD)
GENDER	
Female	127 (42%)
Male	175 (58%)
AGE (years, range 21–65)	35.2 (10.0)
YEARS OF JOB EXPERIENCE	
0–1	36 (11.9%)
2–5	111(36.8%)
6–10	53 (17.5%)
11–20	58 (19.2%)
21–30	29 (9.6%)
>30	15 (5%)
TYPE OF EMPLOYMENT	
Part-time	90 (30%)
Full-time	212 (70%)

The descriptive statistics and Pearson correlations among the variables included in the study are reported in Table 2. All correlations were statistically significant, with the strongest association being between impaired sleep (ISI) and work-related stress (POS) (*r* = 0.38) and the weakest between work-related stress (POS) and substance use (COPE-SUBST) (*r* = 0.12).

**Table 2.** Descriptive statistics and Pearson's correlations among the measures of interest.

Variable	Mean (SD)	Median	Min/max	ISI	POS	CWB-O
ISI	9.28 (5.57)	9.00	0/28	-		
POS	2.69 (0.92)	2.75	1/5	0.38***	-	
CWB-O	1.51 (0.45)	1.38	1/3.38	0.24***	0.26***	-
COPE-SUBST	1.30 (0.69)	1.00	1/4	0.26***	0.12*	0.34***

Note: ISI: Insomnia Severity Index; POS: Perceived Occupational Stress; CWB-O: Counterproductive Work Behavior toward the Organization; COPE-SUBST: Brief COPE Substance Use subscale. * p < 0.05, ** p < 0.01, *** p < 0.001.

A moderated mediation analysis was conducted to test the three hypotheses. The results of the indirect effect of ISI on CWB-O via POS, at three levels of COPE-SUBST (Mean, Mean − 1SD, and Mean + 1SD), are presented in Table 3. At the average level of COPE-SUBST, ISI was found to be a significant predictor of POS (β = 0.37, *p* < 0.001), which in turn was significantly associated with CWB-O (β = 0.18, *p* < 0.001). The direct effect of ISI on CWB-O was not significant (β = 0.10, *p* = 0.08), but the overall indirect (i.e., mediated by POS) effect was significant (β = 0.07, *p* < 0.01), thus supporting H1.

**Table 3.** Mediation effects for different COPE-SUBST moderator levels.

Moderator levels							
COPE-SUBST	Type	Effect	b	95% C.I.	β	z	p
Mean − 1SD	Indirect effect	ISI==>POS==>CWB-O	0.003	−0.001/0.006	0.03	1.53	0.13
Mean − 1SD	Path	ISI==>POS	0.06	0.04/0.09	0.37	4.91	<0.001
Mean − 1SD	Path	POS==>CWB-O	0.04	−0.01/0.10	0.09	1.61	0.11
Mean − 1SD	Direct effect	ISI==>CWB-O	0.01	−0.001/0.02	0.10	1.70	0.09
Mean − 1SD	Total effect	ISI==>CWB-O	0.02	0.01/0.03	0.24	4.31	<0.001
Mean	Indirect effect	ISI==>POS==>CWB-O	0.005	0.002/0.009	0.07	2.91	<0.01
Mean	Path	ISI==>POS	0.06	0.04/0.08	0.37	6.70	<0.001
Mean	Path	POS==>CWB-O	0.09	0.03/0.14	0.18	3.24	<0.001
Mean	Direct effect	ISI==>CWB-O	0.008	−0.001/0.02	0.10	1.74	0.08
Mean	Total effect	ISI==>CWB-O	0.02	0.01/0.03	0.24	4.31	<0.001
Mean + 1SD	Indirect effect	ISI==>POS==>CWB-O	0.008	0.003/0.01	0.10	3.26	<0.001
Mean + 1SD	Path	ISI==>POS	0.06	0.03/0.09	0.37	4.40	<0.001
Mean + 1SD	Path	POS==>CWB-O	0.13	0.08/0.19	0.27	4.87	<0.001
Mean + 1SD	Direct effect	ISI==>CWB-O	0.008	−0.001/0.02	0.10	1.77	0.08
Mean + 1SD	Total effect	ISI==>CWB-O	0.02	0.01/0.03	0.24	4.31	<0.001

Note: ISI: Insomnia Severity Index; POS: Perceived Occupational Stress; CWB-O: Counterproductive Work Behavior toward the Organization; COPE-SUBST: Brief COPE Substance Use subscale.

The strength of the indirect effect of ISI on CWB-O via POS varied depending on the level of COPE-SUBST. At low levels of substance use (Mean − 1SD COPE-SUBST), the indirect effect was not significant (β = 0.03, *p* = 0.13). However, at high levels of substance use (Mean + 1SD COPE-SUBST), the indirect effect was both significant and stronger (β = 0.10, *p* < 0.001) than at average levels of COPE-SUBST (β = 0.07, *p* < 0.01). Furthermore, the effect of POS on CWB-O also varied with levels of substance use: it was not significant at low levels of COPE-SUBST (β = 0.09, *p* = 0.11) but became significant and stronger at high levels of COPE-SUBST (β = 0.27, *p* < 0.001). Notably, the effect of ISI on POS remained consistent across different levels of COPE-SUBST (β = 0.37, *p* < 0.001 at low, average, and high levels).

**Table 4.** Effects of moderation taking into account the relationships of the mediation model.

Moderator	Interaction	b	95% C.I.	β	z	p
COPE-SUBST	ISI: COPE-SUBST==>POS	−0.001	−0.03/0.03	−0.002	−0.03	0.97
	POS: COPE-SUBST==>CWB-O	0.06	0.04/0.09	0.30	5.66	<0.001

Note: ISI: Insomnia Severity Index; POS: Perceived Occupational Stress; CWB-O: Counterproductive Work Behavior toward the Organization; COPE-SUBST: Brief COPE Substance Use subscale.

**Figure 2.**
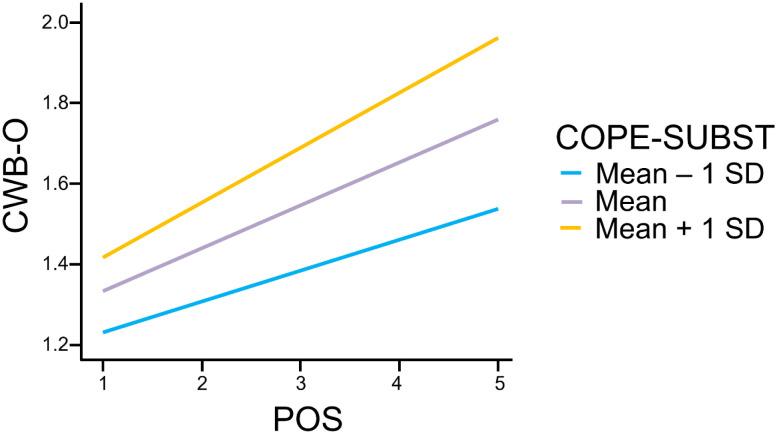
The moderating effect of substance use (COPE-SUBST) on the relationship between work-related stress (POS) and counterproductive work behavior toward the organization (CWB-O).

Table 4 confirms the significant interaction effect between POS and COPE-SUBST on CWB-O (β = 0.30, *p* < 0.001), indicating that the relationship between work-related stress and counterproductive work behaviors directed toward the organization is moderated by the use of substances as a coping strategy. This interaction suggests that as substance use increases, the impact of work-related stress on CWB-O is amplified. To illustrate this interaction effect, the predicted levels of CWB-O at low, medium, and high levels of COPE-SUBST are plotted in Figure 2. This finding supports H2b, demonstrating the conditional effect of substance use on the stress–CWB-O relationship.

Conversely, as reported in Table 4, no significant interaction was found between ISI and COPE-SUBST on POS (β = −0.002, p = 0.97), suggesting that the relationship between impaired sleep and work-related stress does not change based on the level of substance use. Therefore, H2a was not supported.

**Figure 3.**
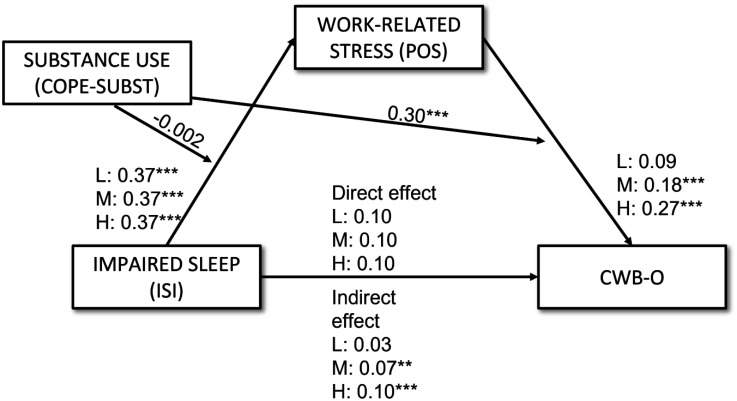
Results of the moderated mediation model (in standardized beta scores) for low (L), medium (M), and high (H) levels of substance use. * p < 0.05, ** p < 0.01, *** p < 0.001.

The standardized coefficients for the individual paths in the moderated mediation model are presented in Figure 3.

## 4. Discussion

The aim of the present study was to explore the complex relationships among sleep problems, work-related stress, and counterproductive work behaviors directed toward the organization, and the moderating role of substance use as a dysfunctional coping strategy. The results contribute to a better understanding of how these factors interact and offer new insights into the mechanisms that underlie behavioral dysregulation at work, especially in situations of resource depletion, in accordance with COR theory [57],[58].

The first hypothesis (H1) stated that work-related stress would mediate the relationship between impaired sleep and CWB-O. The results supported this hypothesis, indicating that sleep difficulties significantly intensify employee's perceived stress at work, which in turn increases the likelihood of engaging in CWB-O. This finding is consistent with COR theory, which states that when essential resources like sleep are depleted, individuals become more vulnerable to stress and, as a consequence, more prone to deviant behaviors [62]. These behaviors, oftentimes aggressive, can, in fact, be a defense strategy taken on to mitigate adverse circumstances and protect whatever resources individuals still have [58]. It is noteworthy that the direct effect of sleep on CWB-O was not significant, indicating that it is the work-related stress increased by sleep impairment, rather than lack of sleep itself, that drives these deviant behaviors.

The second hypothesis (H2a) proposed that the relationship between sleep impairment and work-related stress would be moderated by substance use as a dysfunctional coping strategy. This hypothesis, however, was not supported since the effect of sleep on stress levels remained constant independent of substance use levels. A possible explanation for this result is that the relationship between lack of sleep, substance use, and work-related stress could be more complex than a straightforward interaction. For example, the direct effect of impaired sleep on stress could be so strong that only a very high level of substance use would significantly moderate this relationship [100]. This potential threshold effect could not have been captured by the theoretical model used in this study. Also, we employed a non-clinical sample; therefore, participants may not have engaged in substance use at levels high enough to affect the sleep–stress relationship. Additionally, the tool employed to measure substance use (the Brief COPE) specifically assesses the use of substances as a coping mechanism for stress, which may have limited its ability to capture substance use aimed at coping with sleep problems. Another possibility is that substance use acts as an independent risk factor for sleep impairment, rather than interacting directly with the sleep–stress relationship. Specifically, it could contribute to poorer sleep quality, which, in turn, leads to higher levels of stress [85],[86] in a resource loss cycle [58], where individuals lose multiple resources and over time become increasingly limited in their ability to offset potential strains at work.

Finally, results provided substantial support for the third hypothesis (H2b), which stated that the relationship between work-related stress and CWB-O would be moderated by the dysfunctional coping strategy of substance use. Indeed, higher substance use increases the effect of work-related stress on CWB-O, a relationship that was not even significant for low levels of substance use. This finding aligns with existing literature suggesting that substance use impairs cognitive and emotional functioning, leading to poorer decision-making and reduced self-regulation [91],[92]. Specifically, a possible explanation can be that a maladaptive strategy of coping with work-related stress by substance use, although perceived as helpful in the short term, results in a downward spiral in the long run, in which employees are even more susceptible to experiencing low mood, negative emotions, and negative attitudes toward the stressful environment at work. As a consequence, individuals under stress who engage in substance use are more likely to engage in deviant behaviors at work as a means of defense. Thus, while substance use does not affect the relationship between sleep impairment and work-related stress, it plays a critical role in how work-related stress contributes to dysregulated behaviors at work.

These findings contribute to the growing body of literature on workplace behavioral dysregulation and occupational stress by highlighting the critical role of substance use as a moderator in the stress-behavior relationship. From a theoretical standpoint, this study extends the COR theory by demonstrating that the use of substances as a coping mechanism can amplify the effects of resource depletion, specifically in the context of behavioral dysregulation in the workplace. In practice, these findings suggest that organizational interventions, other than focusing on stress management, should also promote healthy sleep patterns and address substance use as crucial factors in increasing employees' well-being and reducing counterproductive behaviors.

One of the strengths of this study consists of the use of a moderated mediation model, which is able to capture not only direct effects between variables but also conditional and interactive effects. This approach is particularly useful in organizational research because it acknowledges that behaviors and outcomes are rarely the product of a single factor. Rather, they result from the interaction of multiple factors, also including sleep disorders and coping strategies.

Some limitations should also be acknowledged. First, the cross-sectional design of the study limits the ability to draw causal conclusions. Although the theoretical model we developed included specific directional relationships derived from existing literature [21],[73], further longitudinal research is needed to confirm the temporal sequence of these effects. Second, the study sample was drawn from an online survey platform, therefore the generalizability of the findings to broader populations could be limited. Additionally, we did not collect information about the types of jobs participants were employed in, which could provide important context for understanding their stress and coping behaviors. Third, we used the substance use subscale of the Brief COPE because it evaluates the frequency of using substances specifically as a coping mechanism, but it lacks details about the types of substances and their patterns of use. Finally, the use of self-report measures may introduce potential biases, such as common method bias or recall bias, which could affect data accuracy. Future studies should consider using objective measures, for example for assessing the severity of sleep problems and substance use, and exploring the relationship between these factors and work-related stress across diverse job types.

In conclusion, this study contributes to our understanding of the relationship between impaired sleep, work-related stress, and counterproductive work behaviors, considering the effect of substance use, a common dysfunctional coping strategy. By shedding light on the conditions under which work-related stress leads to deviant, dysregulated behaviors in the workplace, this research provides insights for both theory and practice. Through the inclusion of sleep quality programs and support for substance abuse in occupational stress management programs, organizations could at the same time improve employee well-being and reduce the incidence of deviant behaviors at work, ultimately creating a more productive and positive work environment.

## Use of AI tools declaration

The authors declare they have not used Artificial Intelligence (AI) tools in the creation of this article.
